# Quality of life of elementary school students with sleep-disordered breathing and allergic rhinitis: A population-based study in Thailand

**DOI:** 10.1371/journal.pone.0310331

**Published:** 2024-09-11

**Authors:** Paskorn Sritipsukho, Chanapai Chaiyakulsil, Pimchanok Junsawat

**Affiliations:** 1 Faculty of Medicine, Department of Pediatrics, Thammasat University Hospital, Thammasat University, Pathumthani, Thailand; 2 Center of Excellence in Applied Epidemiology, Thammasat University, Pathumthani, Thailand; Helwan University Faculty of Engineering, EGYPT

## Abstract

**Background:**

Sleep-disordered breathing (SDB) and allergic rhinitis (AR) are common problems that can lead to worsening quality of life (QOL) in children with these conditions. There is scarce evidence on the QOL of children with SDB outside of the hospital setting with inconsistent evidence on the association of AR and QOL concerning the SDB in children. Thus, the primary objective of this study is to determine the QOL concerning the SDB of elementary school students by using OSA-18. We also aim to provide the relationship of allergic rhinitis to the QOL.

**Methods:**

A cross-sectional study was conducted on all elementary school students, aged 6–12 years, from 10 elementary schools. The QOL of all participants was evaluated by the Thai version of the caregiver-administered OSA-18 questionnaire. The simple and multiple linear regression models were used to determine the effect of allergic rhinitis on the OSA-18 total scores.

**Results:**

A total of 3,053 children were included in the final analysis, 50.1% male. At least a moderate impact on QOL from SDB was observed in 9.4% of the population. Children with AR had significantly higher mean total OSA– 18 scores than the children without AR (47.5 ± 15.0 VS 38.5 ± 13.1, *p <* 0.001). After the adjustment for age, gender, body mass index, household income, and history of asthma, the point estimate of the adjusted beta regression coefficient on the OSA-18 total score in children with AR was 7.82 (95% CI: 6.00–9.65, *p <* 0.001). Significant associations were observed between AR and all domains except for emotional distress.

**Conclusions:**

A substantial number of elementary school children had at least a moderate impact on the QOL from SDB, especially those with AR. Thus, effective screening of SDB in children with AR should be done to improve the QOL of these children.

## Introduction

Sleep-disordered breathing (SDB), such as snoring and obstructive sleep apnea/hypopnea syndrome (OSAHS) in children is a common problem that pediatricians can encounter in everyday practice. A systematic review by Lumeng et al. in 2008 revealed the overall prevalence of parent-reported snoring at 7.45% with a predilection towards boys than girls, heavier children, and African Americans [1]. In Asia, a prevalence study conducted in Thai school-aged children demonstrated that the prevalence of habitual snoring and OSAHS were 8.5% and 0.69%, respectively [2]. SDB is associated with a wide array of negative consequences, such as behavioral problems, poor academic results, and long-term cardiovascular dysfunction [3–5]. These negative conditions can have a major impact on the quality of life (QOL) of these children. Another everyday common encounter in clinical practice that might be associated with SDB is allergic rhinitis (AR). Sleep disturbances and morning awakening problems are reported in patients with both perennial and seasonal AR [6,7]. By having both AR and SDB, further impairment in QOL might ensue.

The World Health Organization (WHO) defined the health-related quality of life (HRQOL) as an individual’s perception of the impact of the disease and treatment based on the multiple factors surrounding cultures, society, expectations, and concerns [8]. The most commonly utilized instrument to measure the HRQOL of children with OSAHS is OSA-18. OSA-18 was developed and first reported by Franco et al. in 2000 as a brief and reliable questionnaire that is easily administered during patient visits to determine the impact of HRQOL in children with OSAHS [9]. The questionnaire consists of 18 items, scored on a 7-point Likert scale, which can be grouped into 5 domains including sleep disturbance, physical suffering, emotional distress, daytime problems, and caregiver concerns. OSA-18 has been validated and cross-culturally translated into several languages, including the Thai language [10]. A study by Sistla et al. showed an improvement in the QOL of children with SDB due to adenotonsillar hypertrophy by using the OSA-18 questionnaire in both the medical treatment group and the surgical treatment group, with the efficacy of medical treatment in mild-moderate SDB and surgery for severe SDB [11]. Another study in Brazil also showed that performing adenotonsillectomy in children with SDB would improve the QOL as measured by OSA-18 [12].

As of present, most studies measuring SDB-related HRQOL were limited to hospital settings determining the after-treatment impact but scarce evidence in literature was found concerning the SDB-related HRQOL of children in the community setting, especially in South East Asia. Furthermore, the association of AR and SDB-related impaired QOL is not consistent among studies. Due to the high prevalence of both conditions in the community, establishing the relationship would provide great economic and social implications for the improvement of treatment and QOL among patients with these conditions. Thus, the primary objective of this study is to determine the SDB-related HRQOL of elementary school children in the school and community setting by using OSA-18. We also aim to provide the relationship of AR to HRQOL of the participating children.

## Material and methods

A cross-sectional study was conducted on all elementary school students, aged 6–12 years, from 10 elementary schools located in Pathum Thani province, central of Thailand from February 2024 to June 2024. Demographic data, characteristics, and the history of allergic rhinitis and asthma diagnosed by physicians of all participants were collected from their caregivers. The SDB-related HRQOL in all participants was evaluated by the Thai version of the caregiver-administered OSA-18 questionnaire. The total OSA-18 score and each subscale score including sleep disturbance, physical suffering, emotional distress, daytime problems, and caregiver concern were calculated and compared between pupils with and without allergic rhinitis. This study was approved by The Human Research Ethics Committee of Thammasat University (Medicine) MTU-EC-PE-0-257/66. This study sample size of 3,000 pupils is sufficient to determine the minimum effect size of 0.2 (the mean difference of OSA-18 total scores between pupils with and without allergic rhinitis divided by the standard deviation) with the estimated prevalence of allergic rhinitis of 16% [13], alpha error of 0.05 and the power study of 90%. Written informed consent was obtained from all participating individuals and caretakers.

### Statistical analysis

Data were analyzed using STATA for Windows v14.0 (StataCorp LLC, Texas, USA). Categorical variables were reported as frequency and percentage. Continuous data were reported as mean and standard deviation. Chi-squared test was used to test the proportion difference between allergic rhinitis and non-allergic rhinitis groups. Student t-test was used to test the mean difference of continuous data between allergic rhinitis and non-allergic rhinitis groups. A linear regression model was used to determine the effect of allergic rhinitis on the OSA-18 total scores. Adjusted beta coefficients from multiple linear regression models with potential confounders including gender, age, household income, history of asthma, and body mass index were used to determine the adjusted effect of allergic rhinitis on the OSA-18 total scores. The OSA-18 scores of each 5 domains were also analyzed correspondingly.

## Results

A total of 3,283 questionnaires were answered with a response rate of 85.3%. Two hundred thirty questionnaires were excluded due to incomplete data. Thus, approximately 3,053 children were included in the final analysis.

Table 1 demonstrates the characteristics of the participating pupils in the study. Approximately 1,531 children (50.1%) were male with a mean age of 7.02 ± 0.40 years. Mother was the main informant in the study encompassing 65.2% of the answered questionnaires. A majority of the children have a household income between 10,000–50,000 baht per month (270–1360 USD/month) (72.9%). Physician-diagnosed allergic rhinitis and asthma were found in 13.4% and 5.9% of the studied population, respectively.

**Table 1:** Characteristics of pupils.

	Total (n = 3,053)	
	n	%
**Informants**		
1. Mother	1,859	65.2
2. Father	548	19.2
3. Others	446	15.6
**Household income (baht per month)**		
<10,000 (< 270 USD)	556	18.2
10,000–30,000 (270–820 USD)	1,729	56.6
30,000–50,000 (820–1360 USD)	498	16.3
50,000–100,000 (1360–270 USD)	213	7.0
>100,000 (> 2700 USD)	57	1.9
**Age in years, mean ± SD**	7.02 ± 0.40	
**Gender (male)**	1,531	50.1
**Obesity (BMI>25kg/m2)**	128	10.7
**History of allergic rhinitis**	408	13.4
**History of asthma**	180	5.9
**Impact on QOL with SDB** *		
Moderate impact**	272	8.9
Severe impact***	18	0.6

* Health-related quality of life with sleep disordered breathing by OSA-18 questionnaire.

** OSA-18 total scores between 60–80.

*** OSA-18 total scores >80.

USD: United States dollars; SDB: Sleep-disordered breathing.

### Quality of life of the studied population

**Fig 1:**
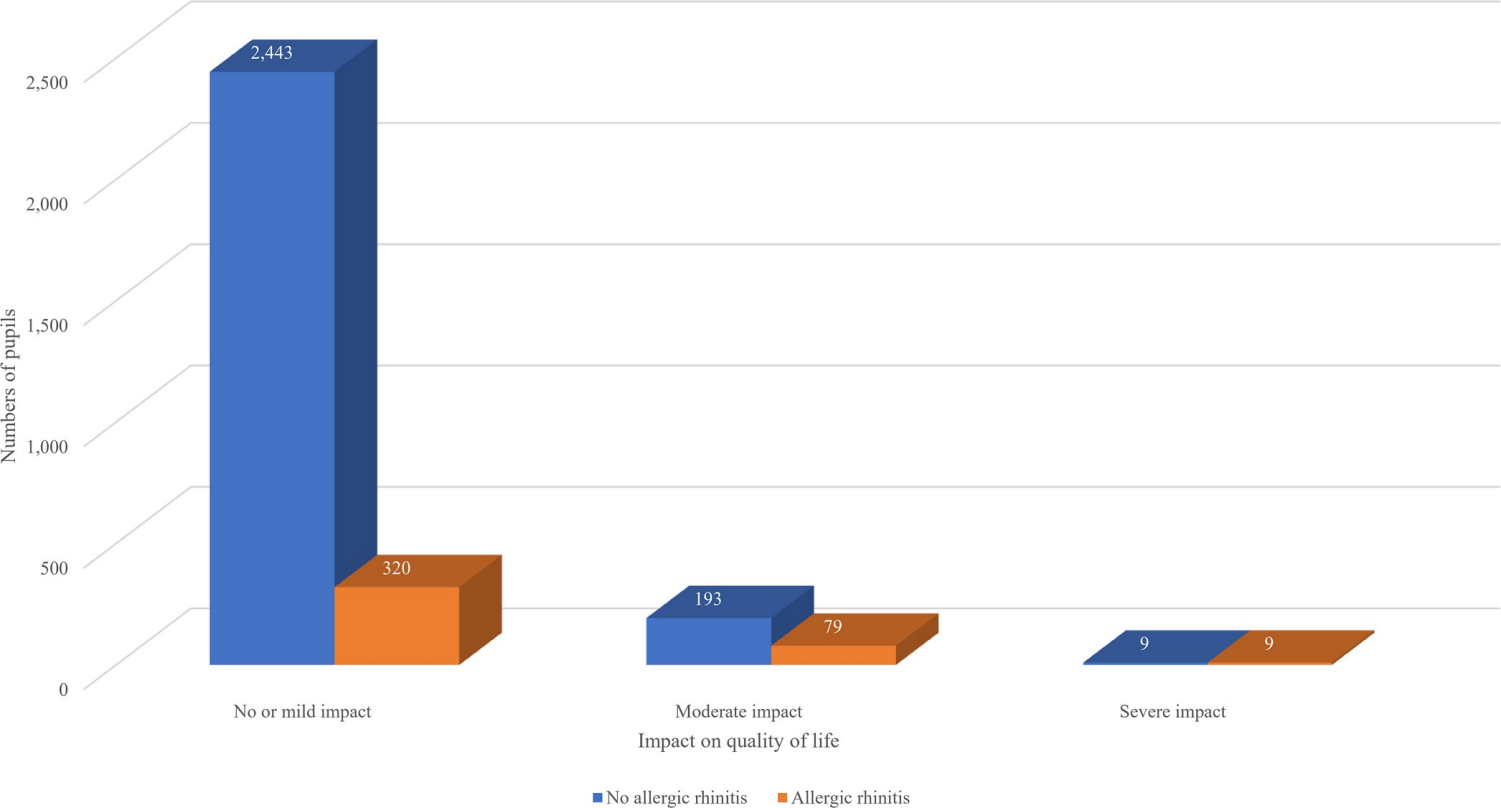
Impact on quality of life with sleep-disordered breathing between children with and without allergic rhinitis. Allergic rhinitis and no allergic rhinitis.

The mean total OSA-18 score among the whole cohort was 39.7 ± 13.8. Moderate impact on SDB-related QOL (OSA-18 60–80) was observed in 8.9% of the population, whilst 0.6% had a severe impact (OSA-18 > 80) [9]. Children with AR had significantly higher mean total OSA– 18 scores than the children without AR (47.5 ± 15.0 VS 38.5 ± 13.1, *p <* 0.001). Furthermore, the scores of children with AR were significantly higher than children without AR in all aspects within the 5 domains of OSA-18 except daytime drowsiness (Table 2). There was a significantly higher proportion of children with at least moderate impact on QOL from SDB in the AR group when compared to the non-AR group (21.6% VS 7.6%, *p <* 0.001) (Fig 1).

**Table 2:** Quality of life (QOL) with sleep-disordered breathing and allergic rhinitis.

Quality of life within the past 4 weeks	Non-allergic rhinitis (n = 2,645)		Allergic rhinitis (n = 408)		p-value
	Mean	Standard deviation	Mean	Standard deviation	
**OSA-18 total scores**	38.5	13.1	47.5	15.0	<0.001
**Sleep disturbance**	6.5	3	8	3.9	<0.001
1. Loud snoring	2.5	1.5	3.1	1.6	<0.001
2. Intermittent breath pauses	1.2	0.7	1.5	0.9	<0.001
3. Choking/gasping during sleep	0.9	1.1	1.3	1.4	<0.001
4. Fragment of sleep	1.9	1.9	2.2	1.3	<0.001
**Physical abnormalities**	8.4	3.6	12.1	4.3	0.004
5. Mouth breathing	1.9	1.3	3.0	1.6	<0.001
6. Frequent colds or upper respiratory tract infection	2.2	1.3	3.6	1.6	<0.001
7. Runny nose	2.8	1.4	3.9	1.4	<0.001
8. Difficulty swallowing	1.4	0.8	1.7	1.1	<0.001
**Emotional distress**	5.5	3.2	6.0	3.7	<0.001
9. Mood swings/ tantrums	1.7	1.1	1.8	1.3	0.008
10. Aggression/hyperactivity	1.9	1.2	2.1	1.4	0.008
11. Discipline problems	2.0	1.3	2.1	1.5	0.024
**Daytime function**	7.1	3.2	7.9	3.4	<0.001
12. Daytime drowsiness	2.2	1.3	2.2	1.3	0.207
13. Trouble concentrating	2.1	1.4	2.6	1.7	<0.001
14. Difficulty awakening	2.8	1.7	3.0	1.8	0.004
**Caregiver concern**	11.1	5.5	13.5	5.8	<0.001
15. Concern about the child’s health	4.4	2.1	5.0	1.9	<0.001
16. Concern about the child’s insufficient breath	2.7	2.0	3.5	2.1	<0.001
17. Impact on work/other daily routine	1.9	1.4	2.4	1.6	<0.001
18. Feeling upset/anxious about the problem	2.1	1.3	2.7	1.7	<0.001

**Fig 2:**
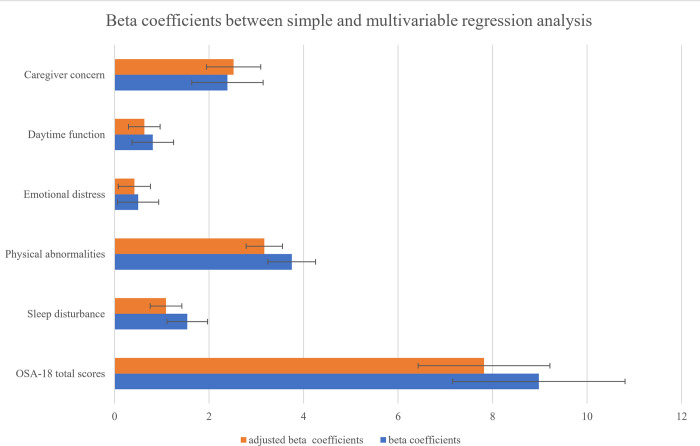
Beta coefficients between simple and multivariable regression analysis. Beta coefficients and adjusted beta coefficients.

Table 3 and Fig 2 illustrate the effect of AR on the QOL in children based on OSA-18. By using simple linear regression analysis, the point estimate of the beta regression coefficient on the OSA-18 total score was 8.98 (95% confidence interval (CI): 7. 59–10.38, *p <* 0.001). There were also significant associations between AR and all five domains within OSA-18. After the adjustment for age, gender, body mass index, household income, and history of asthma, the point estimate of the adjusted beta regression coefficient on the OSA-18 total score in children with AR was 7.82 (95% CI: 6.00–9.65, *p <* 0.001). There were still significant associations between AR and four domains except for emotional distress.

**Table 3:** Effect of allergic rhinitis on the quality of life with sleep-disordered breathing.

OSA-18 scores of each domain	Beta (regression coefficient) ^a^			Adjusted Beta (regression coefficient) ^b^		
	Point estimate	95% Confidence interval	p-value	Point estimate	95% Confidence interval	p-value
**OSA-18 total scores**	8.98	7.59 to 10.38	<0.001	7.82	6.00 to 9.65	<0.001
**Sleep disturbance**	1.54	1.20 to 1.87	<0.001	1.09	0.66 to 1.52	<0.001
**Physical abnormalities**	3.75	3.36 to 4.13	<0.001	3.17	2.66 to 3.67	<0.001
**Emotional distress**	0.5	0.16 to 0.84	0.004	0.42	-0.02 to 0.85	0.062
**Daytime function**	0.81	0.48 to 1.15	0.005	0.63	0.19 to 1.07	0.005
**Caregiver concern**	2.39	1.82 to 2.97	<0.001	2.52	1.77 to 3.28	<0.001

^a^Simple linear regression analysis.

^b^Multiple linear regression analysis: Adjusted for age, gender, body mass index, household income, history of asthma.

## Discussion

In the current study, it was demonstrated that approximately 9.4% of the studied population had at least a moderate impact on SDB-related HRQOL, as defined by OSA-18 > 60 [9]. This was lower than the hospital-based study comparing HRQOL before and after adenotonsillectomy. A study by Silva et al. revealed that 50% of children have a moderate impact on the QOL and 47.9% of children have a severe impact before the surgery [14]. As the current study was a population-based study in the community of elementary school children, a proportion of one-tenth of the children with moderate impact would greatly raise concern about the possible negative consequences in these children with variable access to medical services and different caregiver concerns.

Interestingly, it was also illustrated that children with allergic rhinitis have higher mean OSA-18 scores when compared with children without allergic rhinitis, signifying greater HRQOL impact. Furthermore, there was a significantly higher proportion of at least moderate QOL impact from SDB in children with AR (21.6% VS 7.6%, *p <* 0.001). Despite the adjustment for potential confounders, AR was still found to be associated with all domains of OSA-18 except emotional distress. The hallmark symptoms of AR, such as rhinorrhea and congestion, can lead to worsening sleep quality and daytime sleepiness [15,16]. It was also reported that histamine can serve as a mediator that regulates the sleep-wake cycle [17]. Other inflammatory mediators found in AR such as CysLTs, Interleukin (IL)-1β, and IL-4 are also found to be associated with sleep disturbance and poor sleep quality [18–20]. A recent systematic review and meta-analysis showed evidence of the association between AR and higher risks of insomnia, restless sleep, and SDB, as well as daytime dysfunction [21]. Another recent review article by D’Elia et al. in 2022 describes 23 studies from 2005–2020 aiming to establish the relationship between AR and SDB in children and adolescents. Approximately two-thirds of the studies showed an association between AR and SDB [7]. A study in Thailand by Wongvilairat et al. demonstrated that 23.3% of AR patients are at high risk for obstructive sleep apnea which was two times higher than the general population [22]. Our current study added further evidence to the current literature on the association of AR and SDB, especially in terms of HRQOL.

The major strength of the study is a large, robust sample size. As a population-based study in the community with the data obtained from 10 elementary schools, the generalizability of the population can be better obtained. In contrast with the previous hospital-based studies, a large sample in a population-based study can provide a better picture of the impact of SDB-related HRQOL in the community regardless of the availability of medical access and caregiver concern. Furthermore, with the possible association of two very common conditions such as AR and SDB, especially in terms of HRQOL, greater emphasis should be placed on caring for children with these conditions. The main benefit of a population-based study is the possible recruitment of a large sample of a representative population within the community, significantly reducing the risk of selection bias. The results offer insights into the prevalence and impact of environmental and social factors on health, providing more applicable information to the general population. This contrasts with hospital-based studies focusing on individuals with specific diseases or those seeking healthcare services. Consequently, the findings from hospital-based studies may have limited generalizability due to the specialized nature of the hospital setting and patient population.

This study is not without limitations. The major limitation would be that the current study mainly aims to find the impact of any SDB-related HRQOL based on OSA-18, but not specific to OSA. Nevertheless, with the current study approach, the obtained results could collectively illustrate a broader viewpoint of the HRQOL impact on the lives of children with any type and severity of SDB, not limited to children with OSA. As the population-based study using the questionnaire obtained from the caregivers, no polysomnography was conducted and possible recall bias could occur. Nevertheless, the information obtained within the questionnaire of OSA is sleep-related and daily occurrences which reduces the time lapses between the event and data collection, the errors in recollection could be significantly reduced. Furthermore, parental concerns are also a part of the evaluation within the OSA-18 which has an impact on the QOL of children in parents with high concerns. Moreover, with variable access to PSG in different institutions with different resources, a PSG-based study for the impact of SDB-related HRQOL would greatly limit the sample size of the study and also can lead to potential selection bias of children who have higher parental concerns and better access to medical care.

## Conclusion

A substantial number of elementary school children in the community had at least a moderate impact on the SDB-related HRQOL. An impairment in the HRQOL is significantly higher in children with AR in all aspects except emotional distress. Thus, effective screening of SDB in children with AR should be done to improve the health and well-being of children and their families.
